# Maclise’s Surgical Anatomy

**Published:** 1850-08

**Authors:** 


### MACLISE'S SURGICAL ANATOMY.

We have received from the publishers, Messrs. Lea & Blanchard, the third number of this admirable reprint, containing the Surgical Anatomy of the Inguinal and Femoral Regions most graphically depicted. We are obliged, for want of room, to postpone the critical examination of it until a future number. In the meantime, we feel safe in saying, that it contains the best exposition of the different varieties of hernia that we have ever met with.

